# Senescence in Vascular Smooth Muscle Cells and Atherosclerosis

**DOI:** 10.3389/fcvm.2022.910580

**Published:** 2022-06-01

**Authors:** Yiwen Zha, Wenwen Zhuang, Yongqi Yang, Yue Zhou, Hongliang Li, Jingyan Liang

**Affiliations:** ^1^Medical College, Yangzhou University, Yangzhou, China; ^2^Institute of Translational Medicine, Medical College, Yangzhou University, Yangzhou, China; ^3^Jiangsu Key Laboratory of Integrated Traditional Chinese and Western Medicine for Prevention and Treatment of Senile Diseases, Yangzhou University, Yangzhou, China

**Keywords:** atheroscelorsis, senescence, VSMCs, SASP, sentherapeutic

## Abstract

Vascular smooth muscle cells (VSMCs) are the primary cell type involved in the atherosclerosis process; senescent VSMCs are observed in both aged vessels and atherosclerotic plaques. Factors associated with the atherosclerotic process, including oxidative stress, inflammation, and calcium-regulating factors, are closely linked to senescence in VSMCs. A number of experimental studies using traditional cellular aging markers have suggested that anti-aging biochemical agents could be used to treat atherosclerosis. However, doubt has recently been cast on such potential due to the increasingly apparent complexity of VSMCs status and an incomplete understanding of the role that these cells play in the atherosclerosis process, as well as a lack of specific or spectrum-limited cellular aging markers. The utility of anti-aging drugs in atherosclerosis treatment should be reevaluated. Promotion of a healthy lifestyle, exploring in depth the characteristics of each cell type associated with atherosclerosis, including VSMCs, and development of targeted drug delivery systems will ensure efficacy whilst evaluation of the safety and tolerability of drug use should be key aims of future anti-atherosclerosis research. This review summarizes the characteristics of VSMC senescence during the atherosclerosis process, the factors regulating this process, as well as an overview of progress toward the development and application of anti-aging drugs.

## Introduction

The elderly population is increasing across the world, and vascular aging in particular is a major risk factor for atherosclerotic cardiovascular disease (1). Vascular aging accelerates the progression of atherosclerosis, however, the developmental processes governing atherosclerotic lesions are complex (2). Vascular smooth muscle cells (VSMCs) are located within the vascular architecture and are the predominant cell type involved in all stages of atherosclerotic plaques (3, 4). Cellular senescence has been associated with atherosclerosis development, and can be divided into two discreet categories: replicative senescence (RS) and stress-induced premature senescence (SIPS) (5, 6). Senescent cells cease to proliferate, however, cells remain metabolically active and can promote inflammation, giving rise to the term senescence-associated secretory phenotype (SASP) (7). Senescent VSMCs have been reported in both aged vessels and atherosclerotic plaques (1, 8). The suitability and reliability of traditional markers of senescence have recently been questioned due to a lack of specificity and application across cell types (9–11). Further, existing anti-aging drugs have limitations in their application during atherosclerosis treatment (12). To address these issues in this review, we: (1) attempt to summarize the characteristics of VSMCs senescence during the atherosclerosis process; (2) identify key factors regulating VSMCs senescence; (3) assess potential pathways that may be exploited therapeutically to mitigate VSMC senescence; (4) discuss the progression of atherosclerotic cardiovascular disease, and the current challenges faced in fully characterizing this process.

## Accumulation of Senescent VSMCs During Atherosclerosis

Cellular senescence is not a static cellular state, but a dynamic process during which cells undergo quiescence (initial transient senescence), early senescence (stable growth arrest), complete senescence (chromatin changes associated with senescence and SASP), and late/deep senescence (phenotypic diversification) (13). Similar to other cell types, senescent VSMCs have impaired proliferative potential coupled with increased propensity for expression of cellular senescence markers and cell death (1). VSMCs aging is characterized by a shift from a contractile phenotype to a synthetic phenotype, impaired response to contractile or diastolic mediators secreted by endothelial cells, and changes in ion channel expression and abundance in the cell membrane (14). In addition, the occurrence of senescence follows the formation of VSMC polyploidy. A recent study has shown that proprotein convertase subtilisin/kexin type 9 (PCSK9) induced VSMCs senescence, possibly through a cellular mechanism of polyploidization involving downregulation of apolipoprotein E receptor 2 (ApoER2) (15). In atherosclerosis, senescent VSMCs may be present only in the intima rather than the mesenchyme (16), and VSMCs senescence is associated primarily with plaque size rather than plaque formation (17). Advanced atherosclerotic plaques are covered by fibrous caps containing VSMCs and extracellular matrix (ECM) molecules (18). Given that VSMCs can secrete and deposit ECM proteins, they are generally considered to be protective against atherosclerotic plaque instability (19). However, senescent VSMCs promote plaque vulnerability by secreting matrix-degrading proteases. Compared with normal VSMCs, collagen secretion from senescent VSMCs is reduced which further impairs plaque stability (20). Thus, senescent VSMCs not only accumulate in the atherosclerotic setting, but their properties exacerbate the development of atherosclerosis and increase the risk of atherosclerosis-related complications.

## The Pro-Atherosclerotic Properties of Senescent VSMCs

### Oxidative Stress

**FIGURE 1:**
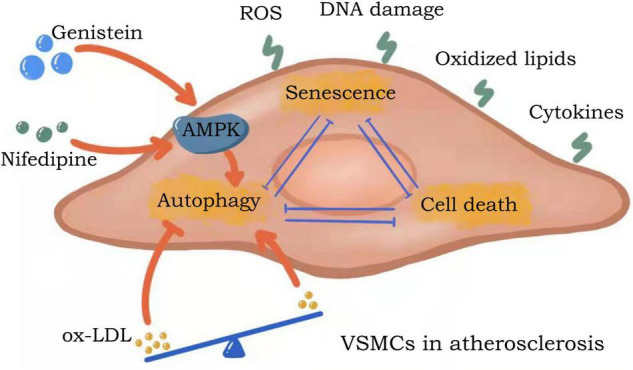
Potential mechanisms of ROS effects on VSMCs senescence and atherosclerosis. Both increased ALHD2 and decreased NOX4 promote ROS production, while vitamin D inhibits ROS production. All of these can regulate the senescence process of VSMCs, thus affecting the formation of atherosclerotic plaques. although oxidative DNA damage occurs in both genomic and mtDNA molecules, the propensity for mtDNA damage due to ROS is disputed. ALDH2, aldehyde dehydrogenase 2; NOX4, nicotinamide adenine dinucleotide phosphate (NADPH) oxidase 4; ROS, reactive oxygen species; mtDNA, mitochondrial DNA.

In conditions considered to be major risk factors for atherosclerotic cardiovascular disease (including diabetes, hypertension, dyslipidemia and smoking), reactive oxygen species (ROS) are at increased abundance in the vessel wall (21). The free radical theory of aging suggests that aging occurs when cells suffer lasting damage due to sustained attack by free radicals (22). Mitochondria and nicotinamide adenine dinucleotide phosphate (NADPH) oxidases (NOXs) are the two major sources of ROS production in the cell (23). Mitochondria are responsible for the production of adenosine triphosphate (ATP) *via* oxidative phosphorylation, a process which produces ROS as a by-product (24). The primary function of NOXs is to produce ROS (23). Alterations in generation of ROS are thought to be important contributing factors to VSMCs senescence and atherosclerotic progression (Figure 1). Studies have confirmed that beneficial effects of the active form of vitamin D on vascular health are achieved by inhibiting the production of free radicals and preventing premature aging of VSMCs (25). Aldehyde dehydrogenase 2 (ALDH2) deficiency in mice can reduce atherosclerotic plaque area by accelerating mitochondrial ROS-mediated VSMC senescence, while promoting plaque instability (26). Overexpression of NOX4 in four-month old mice resulted in elevated mitochondrial H_2_O_2_ and superoxide production in aortic VSMCs, which, in parallel, induced DNA damage and increased cellular senescence and pro-inflammatory gene expression (23).

DNA damage is primarily mediated by physiological or pathological ROS, and the low redox potential of guanine makes this base particularly vulnerable to oxidation (27). 8-Oxoguanine (8oxoG) is the most abundant damaging DNA modification induced by ROS exposure. Human atherosclerotic plaque VSMCs are proposed to have 8oxoG basal excision repair (BER) defects and are associated with reduced 8oxoG DNA glycosylase (OGG1) expression and acetylation (28). BER is a key process in genome integrity and maintenance, evidenced by the severe premature aging and metabolism-deficient phenotypes of BER-deficient mice (29). Notably, although oxidative DNA damage occurs in both genomic and mitochondrial DNA (mtDNA) molecules, the propensity for mtDNA damage due to ROS is disputed (24, 30). When first described, there was no increase in ROS in mutant mice despite the presence of widespread mtDNA deficiency (31, 32). In subsequent studies, no changes in ROS were observed despite increased mtDNA damage, atherosclerosis, and atherosclerotic plaque vulnerability (33). Nevertheless, long-term mtDNA damage leads to mitochondrial dysfunction and more damaging ROS production, which induces cellular senescence and hastens atherosclerosis development (34).

Oxidative stress has also been shown to cause accelerated telomere shortening as it is rich in guanine and therefore prone to prone to be oxidized to 8oxoG (35). Telomere shortening was strongly associated with increasing severity of atherosclerosis, and telomere length was a putative risk factor for atherosclerotic cardiovascular disease (36, 37). Compared with normal VSMCs, human fibrous cap VSMCs exhibited distinctly shorter telomeres (38). Furthermore, loss of telomeric repeat-binding factor-2 (TRF2), which plays an important role in maintaining telomeres, has been shown to promote plaque VSMCs senescence and exacerbate plaque instability in atherosclerosis (8). In addition, increased ROS and SASP have been shown to linked to immune senescence, which could further contribute to increased inflammation and promotes atherosclerosis. In T-Cells, oxidative stress reduced telomerase activity, enabling T-cell senescence and creating pro-inflammatory phenotypes within the plaques, for example (39). However, whether oxidative stress can modulate VSMCs senescence by affecting telomere length and thus the onset and progression of atherosclerosis has not been elucidated.

### Inflammation

A low-grade chronic inflammation which contributes to the development of geriatric disease and persists throughout the geriatric process is known as “inflamm-aging.” Inflamm-aging is caused by the interaction between environmental and genetic factors, and induces a senescent state in VSMCs (40, 41). The aging process in VSMCs is accompanied by SASP, resulting in the production of pro-inflammatory cytokines including, but not limited to, interleukin (IL)-1α, IL-1β, IL-6, IL-8, IL-18 and TNF-α (42). Chronic inflammation is exacerbated by ROS production and reduced antioxidant capacity (43). ROS regulates a variety of cytokines and chemokines at the transcriptional level through modulation of transcription factors, generating a positive feedback loop whereby inflammatory regulators stimulate inflammation and ROS production in the surrounding environment (44). This ultimately leads to an amplification of the aging and atherosclerotic processes.

In the course of atherosclerosis, VSMCs senescence is closely linked to the inflammatory response. Studies suggest that senescent VSMCs may drive the transition to SASP in an IL-1α-dependent manner, inducing adjacent cells into a pro-inflammatory state and thus directly contributing to the chronic inflammation associated with atherosclerosis (45). In human atherosclerosis, coagulation factor Xa (FXa) is locally produced and activated. Chronic stimulation of FXa promotes VSMC senescence, upregulates expression of insulin-like growth factor binding protein 5 (IGFBP-5) and p53, and leads to chronic inflammation (46). Conversely, in mice sirtuin protein 6 is reported to delay the senescence process in VSMCs by promoting stability of atherosclerotic plaques via reduced inflammatory cytokine expression and protection of telomere integrity (47). Several recent studies have reported that alogliptin and omarigliptin, inhibitors of dipeptidyl peptidase-4 (DPP-4), have protective effects against IL-1β and TNF-α-induced VSMC senescence, respectively (48, 49). Therefore, therapies targeting the reduction of inflammation to delay VSMCs senescence may be therapeutically beneficial as anti-atherosclerosis agents to promote plaque stability.

### Calcium Regulators

Vascular smooth muscle cells are key to the vascular calcification process (50, 51). Two broad and discreet vascular calcification processes have been described: arterial medial calcification (AMC) and arterial intimal calcification (AIC)(52, 53). Although the phenotypic alterations associated with calcification in VSMCs are similar for both AMC and AIC, the factors driving calcification in each are significantly different. AMC is commonly seen in patients with diabetes and chronic renal failure (54–56). By contrast, AIC is more often associated with AS, and patients with AIC tend to express high levels of serum pro-inflammatory cytokines despite calcium phosphate homeostasis (57, 58). Genealogy tracking studies in mouse models of atherosclerosis have shown that osteochondrocyte-like precursor cells and chondrocytes observed in atherosclerotic lesions are typically derived from VSMCs, suggesting that these cells are important mediators of AIC (59). Common mechanisms by which VSMCs contribute to AIC may include transdifferentiation into osteochondrocyte and macrophage lineages, facilitating the release of extracellular vesicles and apoptotic vesicles that promote calcification (60, 61), production of collagen and elastin matrices that facilitate calcification (62, 63), and regulation of the production of pro-calcification molecules and inhibitors of calcification (64–66).

Aging VSMCs exhibit a pro-calcification phenotype and activate multiple osteogenic pathways such as runt-associated transcription factor 2 (Runx-2), bone morphogenetic protein 2 (BMP-2), alkaline phosphatase (ALP), osteopontin (OPN), and osteoprotegerin (OPG) in response to stimulation by inflammation and oxidative stress (42, 67). Downregulation of miR-542-3p in VSMCs plays an important role in the osteogenic transformation of these cells in aging rats and is achieved by targeting BMP-7 (68). Recently, myostatin was reported to reduce the expression of Runx-2 and BMP-2 in rat VSMCs, operating through the mammalian target of rapamycin (mTOR) signaling pathway and thereby attenuating VSMCs calcification (69).

The relationship between VSMCs aging and AMC has been elaborated upon by several studies. For example, circulating miR-34a was positively correlated with IL-6 in a healthy population with an age range between 20 and 90 years; while deletion of miR-34a in mice promoted SASP in VSMCs and exacerbation of AMC (70). Furthermore, in a mouse model of aging, senescence was associated with AMC and the appearance of osteoblast-like VSMCs expressing Runx-2 (67). Notably, although aging increases risk of both AMC and AIC, and aging in VSMCs contributes to the pro-calcification phenotypic shift, it is currently unclear whether VSMCs aging is also involved in atherosclerotic AIC.

### Autophagy and Apoptosis

Continuous stimulation of VSMCs in the arterial wall or atherosclerotic lesions by oxidized lipids, ROS, inflammatory cytokines, DNA damage molecules or other stimulants, a response is mounted via three pathways: resistance (autophagy), adaptation (senescence), or death (apoptosis/necrosis) (71–73). VSMCs can promote cell survival by activating autophagy pathways to counteract damage (74). VSMCs adapt to stress conditions by inhibiting proliferation and effectively entering a senescent state. Although VSMCs survive constant stimulation, senescent VSMCs undergo significant metabolic and morphological alterations. When subjected to chronic, excessive stimulation, VSMCs eventually undergo cell death via apoptotic or necrotic pathways.

**FIGURE 2:**
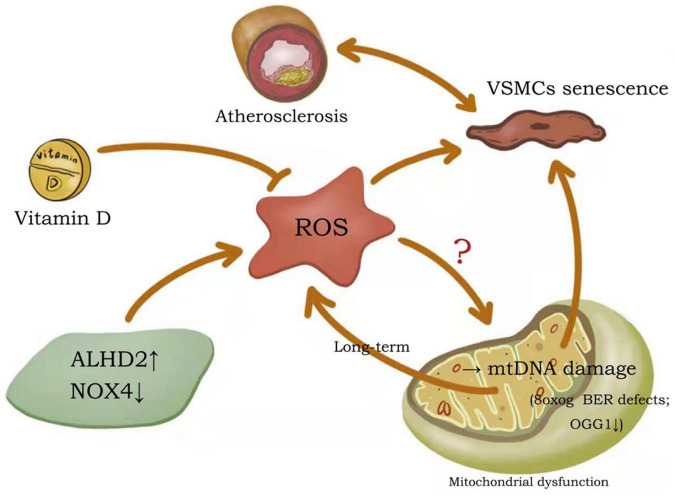
Crosstalk between autophagy, senescence and apoptosis in VSMCs. Continuous stimulation of VSMCs in the arterial wall or atherosclerotic lesions by oxidized lipids, ROS, inflammatory cytokines, DNA damage molecules or other stimulants, a response is mounted via three pathways: resistance (autophagy), adaptation (senescence), or death (apoptosis/necrosis). Autophagy in VSMCs is moderated by a balance of ox-LDL. VSMCs autophagy, senescence and death are interconnected and are negatively correlated in atherosclerosis.

Vascular smooth muscle cells autophagy, senescence, and apoptosis are all interrelated in the context of atherosclerosis (Figure 2). Autophagy in VSMCs is moderated by a balance of oxidized low-density lipoprotein (ox-LDL), where low to moderate ox-LDL concentrations promote autophagy and elevated concentrations inhibit autophagy (75). Stimulation of autophagy inhibits VSMC senescence, and conversely, inhibition of autophagy promotes VSMC senescence. Studies have shown that genistein, the major isoflavone in soy products, can promote autophagy by inducing LKB1-AMPK activation, thereby inhibiting the aging of VSMCs (76). Similarly, nifedipine-induced AMPK activation can inhibit the aging process in VSMCs by regulating autophagic flux (77). Senescent VSMCs are widely characterized as anti-apoptotic due to their expression of anti-apoptotic proteins (78). During apoptosis, the autophagic pathway is inhibited to ensure the complete execution of cell death. Thus, VSMCs autophagy, senescence and death are interconnected and are negatively correlated in atherosclerosis (20).

## Lifestyle Influences VSMCs Senescence

Living a healthy lifestyle may be crucial for retarding aging and preventing age-related diseases. Poor lifestyle practices include smoking and disturbed circadian rhythms, among others. Factors such as the ones listed above may increase cardiovascular risk by negatively affecting VSMCs. More than one billion people worldwide smoke, and smoking accounts for one-third of cardiovascular disease deaths among lifestyle risk factors, and the additional harmful effects of smoking put older adults at higher risk for cardiovascular disease (79). For VSMCs, the source of smoking-induced oxidative stress is NOX-1 (80) and smoking increases the expression of MMP-9. Cotinine, a metabolite of nicotine, has been shown to increase telomerase activity in VSMCs. Thus, even following nicotine metabolism, there can still be long-term adverse effects on vascular function (81). However, whether NOX-1 mediates the effects of nicotine and its metabolites on aging remains to be elucidated. Additionally, circadian rhythms are strongly correlated with atherothrombotic events (82). Researchers have found that circadian misalignment promotes VSMCs apoptosis through defective autophagy (83). However, it is not yet known if circadian rhythms influence VSMCs senescence. To conclude, a healthy lifestyle is almost certainly beneficial to reduce the risk of atherosclerosis, and delaying VSMCs senescence may be one important link of that.

## Senotherapeutics

Pharmacological interventions targeting senescent cells, known as senotherapeutics, fall into two primary categories: senolytics and senomorphics. These agents aim to prevent or delay cellular senescence, thereby delaying age-related pathological processes (84). Senomorphics indirectly prevent cellular senescence by inhibiting SASP, and do not promote cell death (85). Therapies based on senomorphics mainly neutralize SASP by targeting pathways which influence SASP expression (including mTOR, JAK/STAT and PI3k/Akt) and related transcription factors (such as NF-κB and STAT3), or by using targeted antibodies (antibodies targeting IL-1α, IL-6 and IL-8 for example) (84). Previous studies have shown that blackberry, raspberry and black raspberry polyphenol extracts attenuate angiotensin II-induced senescence in VSMCs (86). Recent findings suggest that the atherogenic protective effect of blackberries is mediated through a Nox-1-dependent mechanism, but this is limited to ApoE^–/–^ male mice, not females, and the anti-atherogenic effect of blackberries is independent of circulating total cholesterol, low-density lipoprotein and triglyceride levels (87). Calorie restriction as well as exercise has been shown to be senomorphic. Research has confirmed that calorie restriction without causing malnutrition appears to be the most effective lifestyle-based anti-aging strategy. Calorie restriction reduces risk factors for vascular disease that accompany aging (for example hypertension, glucose/insulin sensitivity and circulating lipid levels) and has direct beneficial effects on vascular oxidative stress resistance and suppression of inflammation (88). Systemic metabolism was altered in calorie-restricted ApoE knockout (ApoE^–/–^) mice, and sirtuin 1 (SIRT1) was significantly upregulated in VSMCs, although the metabolism of VSMCs was not altered. VSMC-SIRT1 is a major sensor of aortic calorie restriction and is associated with matrix metalloproteinase-2 (MMP-2) activation (89). There is growing evidence that sensible and effective exercise regimes improve vascular function and reduce the risk of vascular aging and disease. Telomerase reverse transcriptase (TERT) is the major protein component of telomerase, which extends telomere length and delays cellular aging. Exercise training, an inexpensive lifestyle factor, can increase TERT expression and telomerase activity, thereby reducing telomere wear and tear and effectively extending lifespan (90). In addition, studies have shown that the preventive effect of long-term moderate-intensity continuous training (MICT) on cardiovascular disease is achieved through VSMCs sarc-K_*ATP*_ channels (91).

However, senolytics promote apoptosis in senescent cells (85). Natural compounds quercetin, fisetin and curcumin all belong to the class of senolytics. Natural compounds are mainly found in food and are therefore referred to as “nutraceuticals” (92). Quercetin is a plant flavonoid with powerful antioxidant activity. Fisetin is a bioactive flavonol found in fruits and vegetables, such as apples, strawberries, grapes, onions and cucumbers, with antioxidant activity and anti-inflammatory properties (93). Curcumin, derived from turmeric, has anti-inflammatory, antioxidant, antibacterial and anticancer properties (94). In addition to natural compounds, dasatinib, which is a protein tyrosine kinase inhibitor (95, 96), and ABT-263 and ABT-737, which are BH3-fitted proteins, also belong to the senolytic class of agents (97, 98). There is still uncertainty as to whether senolytics can reduce atherosclerotic plaques by eliminating senescent cells.

Although previous experimental studies have suggested that many anti-aging biochemicals have the potential to treat atherosclerosis, the potential toxic side effects of these drugs should be fully considered before being put into therapeutic application. Senomorphics inhibit SASP but do not permanently eliminate the source of SASP activation, thus requiring chronic or repeated treatment which may increase the incidence of toxic side effects (84). ABT-263 causes transient thrombocytopenia and neutropenia (99). ABT-737 activates a process called minority mitochondrial outer membrane permeabilization (miMOMP), which not only fails to trigger cell death, but also causes DNA damage that promotes tumorigenesis. The above conclusions, however, were drawn only based on *in vitro* experiments, and There is no *in vivo* evidence to support this (100). The possibility of long-term use of these drugs should be considered for patients with atherosclerosis, as a chronic disease. Intriguingly, however, studies have found that the toxicity of senolytics may not represent a clinical problem as short-term treatments may be sufficient for treatment. A treatment regime of 3-days of D&Q once per week over 3 was found to be safe in idiopathic pulmonary fibrosis patients (101). Similar observations were reported in a trial on patients with diabetic kidney disease (102). In view of these facts, such a short-term hit and run approach to senolytic therapy may have the potential to improve tissue function in the long-term while reducing detrimental side effects clinically.

In order to minimize the side effects of such drugs, and to further explore the properties of various senescent cells (including VSMCs) in the pathological process of atherosclerosis, it is necessary to develop delivery systems that can specifically target drugs to the cells of interest. First, it is not currently known at which stage in the disease process do VSMCs express specific senescence markers. VSMCs are not in a static state during the complex atherosclerosis process, and the aging of VSMCs is a multi-step process of changes. Recently, data suggested that traditional cellular senescence markers used to identify senescent VSMCs are no longer expressed during the atherosclerosis process. Therefore, specific, lineage-restricted markers are needed (9). Second, it is unclear whether senolytic drugs prevent atherosclerosis through multiple mechanisms or whether they do so only by clearing senescent cells (9). Not all anti-aging drugs are effective against atherosclerosis; long-term oral administration of dasatinib + quercetin (D + Q) significantly reduced aortic medial senescent cell markers in chronic hypercholesterolemic mice and naturally aging mice, as well as improving vasomotor function, but the mice still developed atherosclerosis. Further, the size of atherosclerotic plaques did not decrease following these treatments (103). Third, before entering the clinical stage of application, optimal conditions for the use of anti-aging drugs should be fully clarified, and the efficacy, specificity and safety of the drugs should be rigourously evaluated.

## Conclusion

Factors associated with the atherosclerotic process, including oxidative stress, inflammation, and calcium-regulating factors, are closely linked to senescence in VSMCs. Despite similarities in characteristic features associated with senescence between VSMCs and other cell types, senescent VSMCs exhibit unique properties during the atherosclerosis process. Promotion of a healthy lifestyle, exploring in depth the characteristics of each cell type associated with atherosclerosis, including VSMCs, and development of targeted drug delivery systems will ensure efficacy whilst evaluation of the safety and tolerability of drug use should be key aims of future anti-atherosclerosis research.

## Author Contributions

YiZ performed the systematic literature search and preparation of the manuscript. JL and HL oversaw the literature review and wrote the manuscript with YiZ. WZ, YY, and YuZ helped with the literature search. All authors contributed to the article and approved the submitted version.

## Conflict of Interest

The authors declare that the research was conducted in the absence of any commercial or financial relationships that could be construed as a potential conflict of interest.

## Publisher’s Note

All claims expressed in this article are solely those of the authors and do not necessarily represent those of their affiliated organizations, or those of the publisher, the editors and the reviewers. Any product that may be evaluated in this article, or claim that may be made by its manufacturer, is not guaranteed or endorsed by the publisher.
